# Improving attitudes toward electroconvulsive therapy

**DOI:** 10.1192/bjb.2021.5

**Published:** 2022-02

**Authors:** Oakley Cheung, Marc Baker, Paul Tabraham

**Affiliations:** 1 Department of Psychology, University of Portsmouth, UK; 2 Portsmouth and South East Hampshire Division, Southern Health NHS Foundation Trust, UK

**Keywords:** Electroconvulsive therapy, patient information, trait empathy, perspective-taking, education

## Abstract

**Aims and method:**

Electroconvulsive therapy (ECT) often causes fear in the general public because of media representation and negative reported side-effects. This study evaluates a new video focusing on experiences of ECT and how this can aid communicating medical information to the public. Knowledge and attitudes toward ECT after watching the video were compared with a group that received no information and a group that read the current NHS leaflet on ECT. The role of empathy was also considered as a covariate.

**Results:**

The video was the only condition found to positively affect knowledge and attitudes toward ECT. The video was especially beneficial to those that possessed low perspective-taking trait empathy.

**Clinical implications:**

These findings demonstrate the video improved knowledge and attitudes toward ECT compared with current material or no information. We suggest that the addition of personal experiences to public information adds perspective, improving overall attitudes toward health treatments.

Electroconvulsive therapy (ECT) is a mental health treatment involving the use of electric currents to induce a seizure,^1^ and is used to treat life-threatening depression, catatonia and severe, long-lasting mania that is resistant to medication.^1,2^ However, ECT raises fear among the general public,^3–5^ largely because of its dramatization in the mainstream media.^2,5–8^ Concerns are, however, not entirely based on fiction, as some patients have reported adverse psychological side-effects as a result of ECT treatment, implying possible signs of trauma response.^9^ The National Health Service (NHS) information for patients undergoing ECT consists of a science-based leaflet from the National Institute for Health and Care Excellence (NICE),^1^ despite research suggesting that healthcare education may be better received alongside contextual and emotive evidence.^9,10^ Gold Coast Health in Australia implemented these principles, focusing on adding contextual evidence for the benefits of ECT,^11^ but there is no empirical evidence as to whether this approach improved perceptions of ECT.

From a psychological perspective, trait empathy is one factor which may affect someone's ability to relate to the personal accounts of those receiving ECT. Both cognitive and affective empathy have a positive relationship with the ability to perceive emotional content.^12^ Specifically, cognitive empathy can facilitate ‘perspective-taking’ capabilities.^13^ Following the use of the video produced by Gold Coast Health,^11^ the Southern Health NHS Foundation Trust developed a similar tool to foster more accurate perceptions of ECT. This study is the first to empirically test whether the use of the emotion-based information improves perception of ECT compared with the current leaflet. We aimed to test whether the new video fulfils its purpose to promote a more positive attitude and better knowledge of ECT in the UK public, and whether trait empathy plays a role in any effects.

## Method

### Ethics

Ethical approval was given by the University of Portsmouth Undergraduate and Taught Postgraduate Psychology Department Research Ethics Board (clearance number 2019-036).

### Design

The experiment used an independent groups design. Participants were randomly allocated to one of three conditions based on type of ECT information (no information, science-based leaflet or emotional video). The dependent variables were mean scores on the ECT knowledge and attitude questionnaire. Two subscales of the Interpersonal Relativity Index (IRI), perspective-taking and empathic concern, were used to control for differing levels of trait empathy in the participant sample.

### Participants

Participants were recruited by volunteer sampling. Posters were advertised around the University of Portsmouth, asking for volunteers to complete an online survey about ECT; these were not aimed at psychology students, but they were not excluded from taking part. Participants that were not students were recruited by sending a request for participation to local community facilities. The survey link was further sent by the non-student participants to their own colleagues, to increase the number of non-student participants. Participant ages ranged from 18 to 67 years (*N* = 146, mean age 31.94 years, s.d. 13.49). The sample comprised 51 men and 95 women, and both students (*n* = 39) and non-students (*n* = 107). Table 1 shows the mean age and gender and student ratios for each condition.

**Table 1:** Participant demographic information

Condition	Mean age in years (s.d.)	Female:male participants	Students:non-students
No information	34.40 (14.37)	29:23	10:42
NHS leaflet	30.98 (12.74)	36:24	17:34
New video	29.98 (13.24)	29:13	11:31

Participants were asked to disclose any experience with ECT, and any mental illness that might qualify an individual for ECT. Some participants disclosed previous experience with ECT (*n* = 64), with a significant proportion (*n* = 21) receiving their knowledge from film or television. Some participants disclosed previous experience of mental health problems (*n* = 84), with the majority (*n* = 63) having experience with severe clinical depression.

### Materials

The survey consisted of a revised version of the IRI,^14^ an ECT attitude and knowledge questionnaire^15^ and two types of information on ECT use as treatment for mental illness.

#### IRI

Two subscales from the revised version of the Basic Empathy Scale^14^ were used to measure trait empathy: empathetic concern and perspective-taking. The scales were rated using five-point Likert scales, with a high score representing higher trait empathy scores.

#### ECT attitude and knowledge questionnaire

The ECT scale consisted of both an attitude and knowledge subscale. Each statement was scored on a six-point scale, with high scores indicating a more positive attitude or correct knowledge of ECT.

#### Information on ECT use in mental health treatment

A public information leaflet was taken from the NICE guidance for the prescription and administration of ECT in depression, mania and catatonia.^1^ This leaflet contains mainly scientific evidence of the efficacy and use of ECT. For this study, the sections ‘What is NICE?’ and ‘What are depressive illness, mania, schizophrenia and catatonia?’ were omitted. A video intervention was developed by the lead author and Southern Health NHS Foundation Trust based on a successful educational video created by Gold Coast Health, Australia.^11^ The video focuses on the experiences of patients, nurses and caregivers with ECT. The video is available to the public at: https://vimeo.com/369525494.

### Procedure

All participants completed the survey online and provided written informed consent before taking part. Participants were first asked about any experience with ECT and any mental health conditions, and then completed the IRI. They were then randomly allocated to one of three information conditions: no information, the current NHS leaflet or the newly developed video. Participants in the leaflet and video condition were presented with the educational material and asked to watch/read carefully before completing the knowledge and attitudes to ECT questionnaire. Participants in the no information condition only completed the knowledge and attitudes to ECT questionnaire.

## Results

### Descriptive statistics

**Fig. 1:**
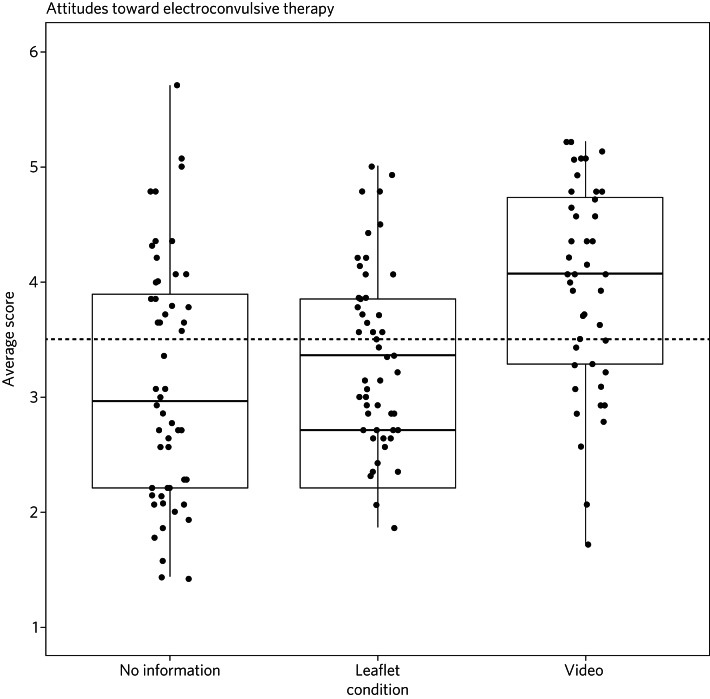
Distribution of electroconvulsive therapy knowledge scores across each information condition (points represent individual participant ratings).

**Fig. 2:**
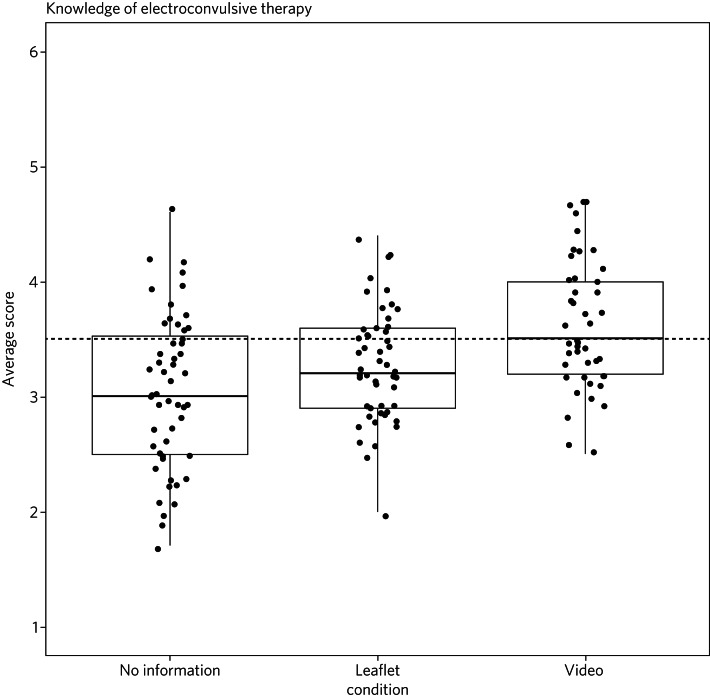
Distribution of electroconvulsive therapy attitude scores across each information condition (points represent individual participant ratings).

Figures 1 and 2 show the distribution of the knowledge and attitude scores in each of the three information conditions. The median scores indicate participants in the video condition had more correct knowledge and a more positive attitude. Importantly, in the attitudes to ECT factor only the video condition showed a median score above the mid-point, indicating a positive attitude. Receiving no intervention or the ECT leaflet had distributions that sat mostly below the mid-point for both knowledge and attitudes, thus meaning the video condition was the only form of intervention likely to encourage accurate knowledge and facilitate a more positive attitude toward ECT when compared with receiving no information or the current leaflet. These findings suggest that video information is the most positive tool of the three studied.

### The role of empathy in the success of the ECT educational material

To analyse the data further, a one-way multivariate analysis of covariance (MANCOVA) was conducted on ECT attitude and knowledge data comparing data from each information condition. Empathetic concern and empathic perspective-taking used as covariates. A significant multivariate effect of information type was found (Wilks’ λ = 0.84, *F*(4, 280) = 6.36, *P* < 0.001, 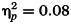), with a small effect size. Empathic perspective-taking was a significant covariate in the multivariate model (Wilks’ λ = 0.95, *F*(2, 140) = 3.44, *P* = 0.035, 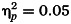), with a small effect size. No significant effect of empathetic concern was found on perception of ECT.

The significant omnibus MANCOVA justified separate univariate ANOVA on the dependent variables. There was a significant effect of information type on knowledge scores (*F*(2, 141) = 11.68, *P* < 0.001, 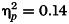), with a small effect size. *Post hoc* pairwise comparisons with a Bonferroni adjustment revealed that knowledge scores were significantly higher in the video condition compared with the leaflet condition (*P* = 0.01) and receiving no intervention (*P* < 0.001). There was also a significant effect of information type on attitudes to ECT scores (*F*(2, 141) = 11.45, *P* < 0.001, 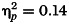), with a small effect size. *Post hoc* pairwise comparisons with a Bonferroni adjustment showed attitude scores to be highest in the video condition when compared with the leaflet condition (*P* = 0.003) and receiving no intervention (*P* < 0.001). There was no significant difference observed between the leaflet and receiving no information for either knowledge or attitudes to ECT.

**Fig. 3:**
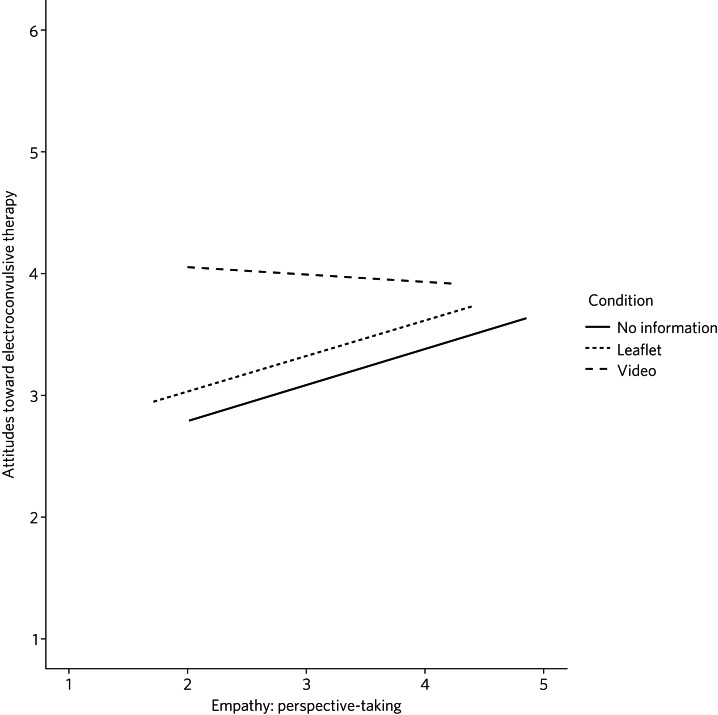
Relationship between perspective-taking and attitudes toward electroconvulsive therapy in each of the information conditions.

Perspective-taking was found to be a significant covariate for attitudes to ECT only (*F*(1, 141) = 5.06, *P* = 0.026, 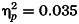), with a small effect size. This suggests the increase in positive attitude to ECT after watching the video exists after controlling for the underlying trait empathy. It also suggests empathic perspective-taking accounts for a very small but significant portion of the variance in attitudes to ECT. Figure 3 shows the relationship between emotional perspective-taking on ECT attitudes in each of the three conditions. Although the regression line for the video was similar across the range of perspective-taking scores (β = −0.06, s.e. 0.25), there was a small positive relationship for the NHS leaflet group (β = 0.29, s.e. 0.16) and the no information group (β = 0.30, s.e. 0.21). When perspective trait empathy was high, attitudes toward ECT in the three information conditions were similar; when perspective trait empathy was low, attitudes toward ECT scores in the new video condition were higher than the NHS leaflet and no information conditions. From this, we can infer that the style of intervention had little effect on those already high in trait empathy; however, for those with low trait empathy, the video proved beneficial for improving perceptions of ECT.

### The role of gender and student status on the effect of ECT educational material

To check the data for potential biases arising from gender and student status, an independent group MANCOVA was conducted on ECT attitude and knowledge scores comparing data from each information condition. Gender (male/female) and student status (student/non-student) were used as covariates. A significant multivariate effect of information type was found (Wilks’ λ = 0.87, *F*(4, 280) = 5.10, *P* = 0.001, 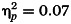), with a small effect size. Gender was a significant covariate (Wilks’ λ = 0.92, *F*(2, 140) = 6.19, *P* = 0.003, 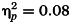), with a small effect size. No significant effect of student status was found on perceptions of ECT. The significant omnibus MANCOVA justified separate univariate analysis of variance on the dependent variables. There was a significant effect of information type on knowledge scores (*F*(2, 141) = 2.88, *P* < 0.001, 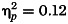), with a small effect size. *Post hoc* pairwise comparisons with a Bonferroni adjustment revealed that knowledge scores were significantly higher in the video condition compared with the leaflet condition (*P* = 0.01) and receiving no intervention (*P* < 0.001). There was also significant effect of information type on attitudes to ECT scores (*F*(2, 141) = 8.87, *P* < 0.001, 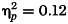), with a small effect size. *Post hoc* pairwise comparisons with a Bonferroni adjustment showed attitude scores to be highest in the video condition when compared with the leaflet condition (*P* = 0.005) and receiving no intervention (*P* < 0.001). There was no significant difference observed between the leaflet and receiving no information for either knowledge or attitudes to ECT. Gender was found to be a significant covariate for knowledge of ECT (*F*(1, 141) = 12.38, *P* = 0.001, 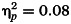), with a small effect size, and attitudes to ECT (*F*(1, 141) = 6.35, *P* = 0.013, 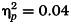). This suggests that the increase in knowledge and a more positive attitude to ECT after watching the video exists after controlling for gender. It also suggests gender accounts for a small but significant portion of the variance in knowledge and attitudes toward ECT.

## Discussion

### ECT knowledge and attitudes

Our findings suggest that both knowledge and attitudes to ECT can be significantly improved using contextual and emotive information. Only the video condition improved knowledge and attitudes to ECT, whereas the leaflet currently used by the NHS did not improve either knowledge or attitudes compared with no information. For both the no intervention condition and the leaflet condition, participants sat below the mid-point for attitudes and on or below the mid-point for knowledge, suggesting that they were still inclined to perceive ECT negatively. These findings suggest that, compared with other styles of intervention, the video would work best to educate patients and carers on the use of ECT to treat mental health illnesses. These results support current literature which suggests that interventions focusing on more emotional, real-life experience may be more effective for perception improvement than using factual information alone.^13,15,16^ It should be noted that all the main and covariate effects were small, and the distributions in all three experimental conditions had participants that perceived ECT both positively and negatively. This suggests that although the video may help to improve perceptions of ECT, it is not a ‘silver bullet’, and might best used alongside other informational material. Future research should assess whether combining the leaflet and the video improved the perceptions above and beyond the video alone.

An alternate explanation for our results may be the modal differences between video and written information; the introduction of a dynamic stimulus may have been enough to demand more attention from participants than reading a leaflet. Some research has suggested showing patients a video can reduce anxiety around healthcare treatments more than written information.^17^ To address this, it would be important to examine whether a similar improvement in knowledge and attitudes is found irrespective of how the content was delivered. It should also be noted that although the efficacy of ECT is outside of the scope of this research, there is still large debate as to whether there are any noticeable and long-lasting benefits to undergoing ECT.^2,3,7,18^ Additionally, meta-analyses report high relapse rates among many patients.^19^ There are some ethical considerations on whether improving attitudes toward ECT is acceptable if the benefit of the treatment is, in some cases, limited and relapse is likely.

### The role of trait empathy

We hypothesised that trait empathy would offer some explanation as to why emotional content was more effective compared with scientific information. We found that perspective-taking influenced attitudes to ECT, but this was only the case for participants who received no information or the NHS leaflet; those with higher perspective-taking trait empathy had a more positive attitude to ECT. Perspective-taking had no effect on attitudes to ECT in the video condition. Therefore, participants with high perspective-taking scores had similar attitudes to ECT in all three conditions, whereas participants with lower perspective-taking empathy had a more negative perception of ECT in the leaflet and no information groups compared with the video group. The video, therefore, seemed to directly improve the attitude of participants who had lower perspective-taking abilities. The proposed reason for this is that the video directly adds context to ECT as a treatment. This allowed participants with lower perspective-taking empathy to relate to the treatment or participants in a similar way to those participants with high perspective-taking empathy.

This explanation seems to be consistent with evidence from neuroimaging studies, which has demonstrated a link between perspective-taking ability and the ventromedial prefrontal cortex,^20^ a brain area that is also critical for perception and reaction to the suffering of others.^19^ Thus, scoring higher in perspective-taking may make an individual more likely to be able to imagine the suffering of those experiencing severe mental health problems, which explains why they may react more positively to ECT even with limited information around the treatment. Furthermore, adding context in the form of another person's account can elicit a more empathetic response from participants when making decisions,^21^and that the empathy elicited is generally more appropriate when context is present;^22^ this suggests that the context in the video may have encouraged a more empathetic response to the content, even for those who do not naturally empathise with another's situation

Alongside the significant covariate effect of trait empathy, gender was found to be a separate significant covariate for both knowledge and attitudes. We suspect that the known gender variation in empathy^23^ can partly explain why gender was a significant covariate. This information provides grounds to suggest further research is conducted into the effect of an emotional, video-based stimulus, and whether any specific gender effects exist in relation to the efficacy of these training materials.

### Clinical implications

These findings provide a deeper insight into the use of education to improve perception of ECT, with emotional stimuli proving to be the best method for information delivery, especially for people with low perspective-taking empathy. Overall, better knowledge of people's experiences with ECT may ultimately mean less fear and apprehension among the public.^24^ The results of our findings can be used as a recommendation for both the NHS and the wider health sector on how to structure and deliver their informational material. A critical point seems to be that personal accounts and context are important in the effective delivery of health information.

### Limitations and suggestions for future research

This study focuses on ECT, which carries a large amount of stigma.^6,8^ Going forward, it could be interesting to explore whether the effect exists with other health treatments with potential negative public perceptions. Some alternative treatments still have stigma attached,^4^ and contextual evidence may be the key to improving perceptions of these treatments for mental health illnesses. The results provide grounds to recommend that more emotive content should be introduced when educating the public about mental health. Suffering from a mental health disorder can still affect your ability to find work and maintain relationships.^25^ Further, certain disorders, such as schizophrenia and psychosis, are still hugely feared by the general public.^26^ Introducing a context and personal experiences to these illnesses helps to distinguish between mental health in the real world and the overdramatization of disorders fed to the public by the media.^27^ Making the distinction between individual and symptom could help integration in society, improve quality of life and aid recovery for those with a mental illness.

## Data Availability

This study was preregistered on 6 November 2019 on the Open Science Framework. Data and details of the preregistration are available at the following link: https://doi.org/10.17605/OSF.IO/SY6AP.

## About the authors

**Oakley Cheung** is a psychology student at the Department of Psychology, University of Portsmouth, UK; **Marc Baker** is a lecturer at the Department of Psychology, University of Portsmouth, UK; **Paul Tabraham** is Consultant Clinical Psychologist and Divisional Lead for Psychological Therapies at the Portsmouth and South East Hampshire Division, Southern Health NHS Foundation Trust, UK
