# Improving attitudes toward electroconvulsive therapy – CORRIGENDUM

**DOI:** 10.1192/bjb.2022.46

**Published:** 2022-12

**Authors:** Oakley Cheung, Marc Baker, Paul Tabraham

**Keywords:** Electroconvulsive therapy, patient information, trait empathy, perspective-taking, education

The authors would like to correct Figures 1 and 2 as well as their captions in the above paper. The correct figures and corresponding captions are included below:

**Fig. 1:**
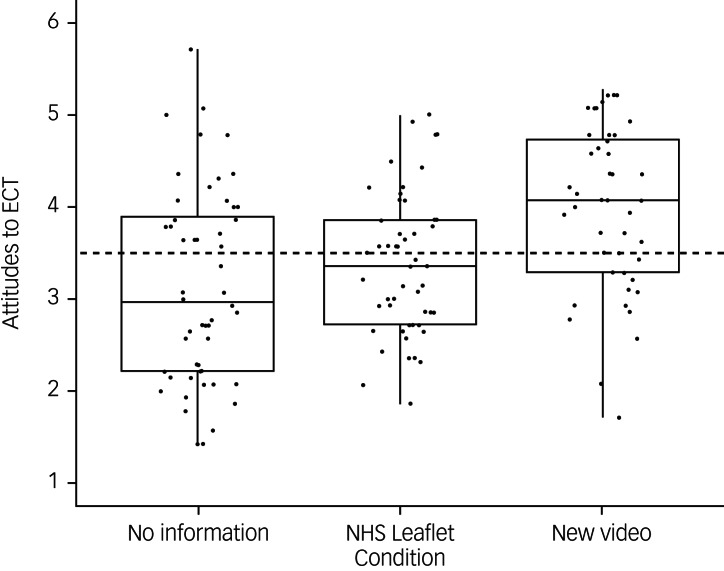
Distribution of electroconvulsive therapy attitude scores across each information condition (points represent individual participant ratings).

**Fig. 2:**
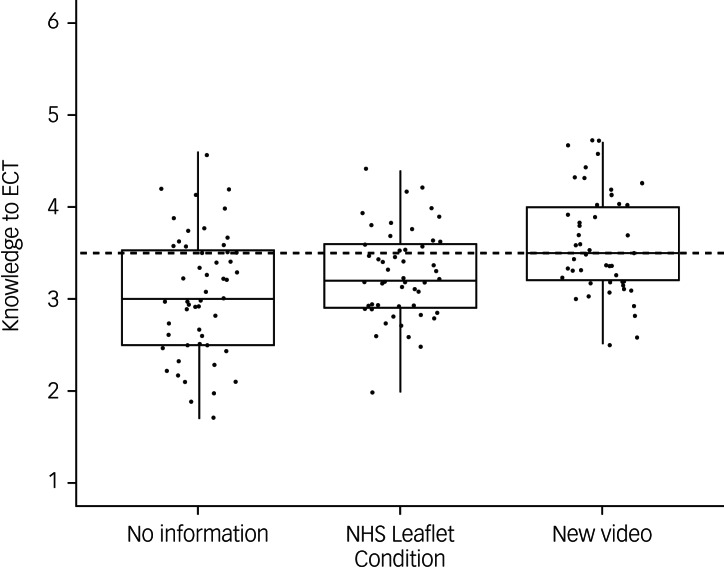
Distribution of electroconvulsive therapy knowledge scores across each information condition (points represent individual participant ratings).

The authors apologise for this error.
